# Neutrophil to lymphocyte ratio in parkinson’s disease: a systematic review and meta-analysis

**DOI:** 10.1186/s12883-023-03380-7

**Published:** 2023-09-21

**Authors:** Samaneh Hosseini, Nasim Shafiabadi, Monireh Khanzadeh, Arshin Ghaedi, Raziyeh Ghorbanzadeh, Amir Azarhomayoun, Aida Bazrgar, Jalil Pezeshki, Hanieh Bazrafshan, Shokoufeh Khanzadeh

**Affiliations:** 1 Neurosciences Research Center, Tabriz University of Medical Sciences, Tabriz, Iran; 2 Mashhad University of Medical Sciences, Mashhad, Iran; 3 Geriatric & Gerontology Department, Medical School, Tehran University of medical and health sciences, Tehran, Iran; 4 Student Research Committee, School of Medicine, Shiraz University of Medical Sciences, Shiraz, Iran; 5 Department of Psychiatry, School of Medicine, Iran University of Medical Sciences, Tehran, Iran; 6 Sina trauma and surgery research center, Tehran University of medical sciences, Tehran, Iran; 7 Shiraz University of Medical Sciences, Shiraz, Iran; 8 Clinical Neurology Research Center, Shiraz University of Medical Sciences, Shiraz, Iran; 9 Tabriz University of Medical Sciences, Tabriz, Iran

**Keywords:** Neutrophil to lymphocyte ratio, NLR, Parkinson’s disease, Meta-analysis

## Abstract

**Background:**

The goal of this research was to explore the role of Neutrophil to lymphocyte ratio (NLR) in Parkinson’s disease (PD).

**Methods:**

From inception to 4 June 2023, PubMed, Web of Science, and ProQuest were searched for papers comparing NLR in PD to healthy individuals. Standardized mean difference (SMD) with a confidence interval (CI) of 95% were calculated.

**Results:**

A random-effect model revealed that PD patients had elevated NLR values compared to healthy individuals (SMD = 0.81, 95% CI = 0.47 to 1.14, P < 0.001). The results of subgroup analysis were as follows: (1) study design: We observed that patients with PD had higher levels of NLR than healthy controls in either retrospective (SMD = 1.12, 95% CI = 0.58 to 1.66, P < 0.001) or prospective (SMD = 0.43, 95% CI = 0.18 to 0.68, P = 0.001) studies. (2) Ethnicity: We noticed that individuals with PD had higher levels of NLR than healthy controls, whether they were East Asian (SMD = 0.93, 95% CI = 0.22 to 1.63, P = 0.010) or Caucasian (SMD = 0.75, 95% CI = 0.40 to 1.10, P < 0.001).The pooled sensitivity of NLR in the prediction of PD was 0.67 (95% CI = 0.61–0.73), and the pooled specificity was 0.66 (95% CI, 0.61–0.70).

**Conclusions:**

Increased levels of NLR is highly related with the presence of PD. Further research is needed to determine the potential clinical benefits of this simple and low-cost biomarker in the PD diagnosis.

**Supplementary Information:**

The online version contains supplementary material available at 10.1186/s12883-023-03380-7.

## Background

Parkinson’s disease (PD) is the second most common neurological illness after Alzheimer’s disease, affecting approximately 7 to 10 million individuals globally [1, 2]. The disease’s most apparent symptoms are related to movement, such as rigidity, movement slowness, tremor, and postural instability [3, 4]. Despite the fact that PD is identified by the appearance of intraneuronal proteinacious cytoplasmic inclusions defined as Lewy bodies and a considerable decrease of dopaminergic neurons in the substantia nigra pars compacta, the illness’s origin remains unknown [5–7]. The antiparkinson medicines, including dopamine agonists and levodopa are often used to treat PD, and they are clinically beneficial in the early stages of disease. However, as the condition worsens, these treatments become ineffective and have adverse side effects [8, 9]. As a result, there is an urgent need to comprehend the etiology of PD and create innovative medicines to prevent or halt disease development.

Inflammation appears to play a prominent role in the pathologic characteristics and symptoms of PD, according to growing research [10–15]. Interleukins and tumor necrosis factors (TNFs) are crucial immune activation signaling molecules that affect both the brain and the periphery [16]. In vivo findings of positron emission tomography and postmortem in PD patients revealed elevated inflammatory reactions, such as microglial activation [17, 18], and higher levels of immunological markers in the brain [18–20].

In addition, several studies have compared inflammatory biomarkers in individuals with PD to healthy controls in an effort to better understand the origin of the condition and identify potential biomarkers for it [21–24].

Neutrophil to lymphocyte ratio (NLR) is a biomarker based on Complete blood count (CBC), and it shows the balance between immunity and systemic inflammation. Although several new studies found links between PD and NLR, the findings were contradictory [25–43]. A meta-analysis on this topic is needed to resolve clinical evidence discrepancies. As far as we know, this is the first systematic review and meta-analysis on this matter.

## Methods

### Study design

We followed the most recent methodological suggestions provided by the Preferred Reporting Items for Systematic reviews and Meta-Analyses (PRISMA) to conduct this meta-analysis [44]. We registered our study in PEROSPERO (CRD42023429384).

### Eligibility criteria

The following PICO terms served as our basis for inclusion:

- Population: Patients suffering from PD.
- Intervention: NLR.
- Control: Healthy controls.
- Outcomes: The diagnostic value of NLR.
- Study design: case-control, cross-sectional, and cohort articles.

Conference abstracts, basic science investigations, animal studies, case reports and case series were all excluded.

### Search strategy

We searched major data bases like Web of Science, ProQuest, and PubMed from inception until June 4, 2023. We used an extensive list of keywords related to NLR and Parkinson’s disease in our search strategy. We did not impose any language or publishing time constraints.

### Study selection

Two review writers independently screened the titles, abstracts, and full texts of publications that appeared in our search to determine which met the criteria for inclusion. If there were any issues, a writer would step in and settle it. Following that, the reference and citation lists of the included papers were examined for further possibly relevant publications.

### Data extraction and management

The extracted information from the selected articles are as follows:

Name of the first author, location, publication year, design of the study, demographic characteristics of the population, mean and standard deviation (SD) of the NLR, cut off point, specificity and sensitivity as effect measures.

### Assessment of methodological quality

Two review authors independently assessed the methodological quality of the included papers using the Newcastle-Ottawa scale (NOS) [45].

### Statistical analysis and data synthesis

Stata 11.2 (Stata Corp, College Station, TX) was employed to conduct the meta-analysis. To compare the NLR values between PD patients and controls, we employed standardized mean difference (SMD) with a 95% confidence interval (CI). To assess the heterogeneity of the included articles, the I^2^ and Cochran’s Q tests were used. Significant heterogeneity across included articles was noted as I^2^ > 50% and Q test p-value < 0.05. We also utilized the random-effects model to assess pooled effects since we noticed a substantial level of heterogeneity. We used the “metandi” command to measure negative likelihood ratio, positive likelihood ratio, diagnostic odds ratio (DOR), and pooled sensitivity and specificity of NLR for PD. In sensitivity analysis, we used “metaninf” command to investigate the influence of every single study on the overall meta-analysis estimate. We also prepared a summary receiver operating characteristic (SROC) curve. Finally, we employed the Egger test and the funnel plot to assess publication bias. GRADE (Grading of Recommendations Assessment, Development and Evaluation) approach was used to assess the certainty of evidence [46].

## Results

### Search results and included studies

**Fig. 1:**
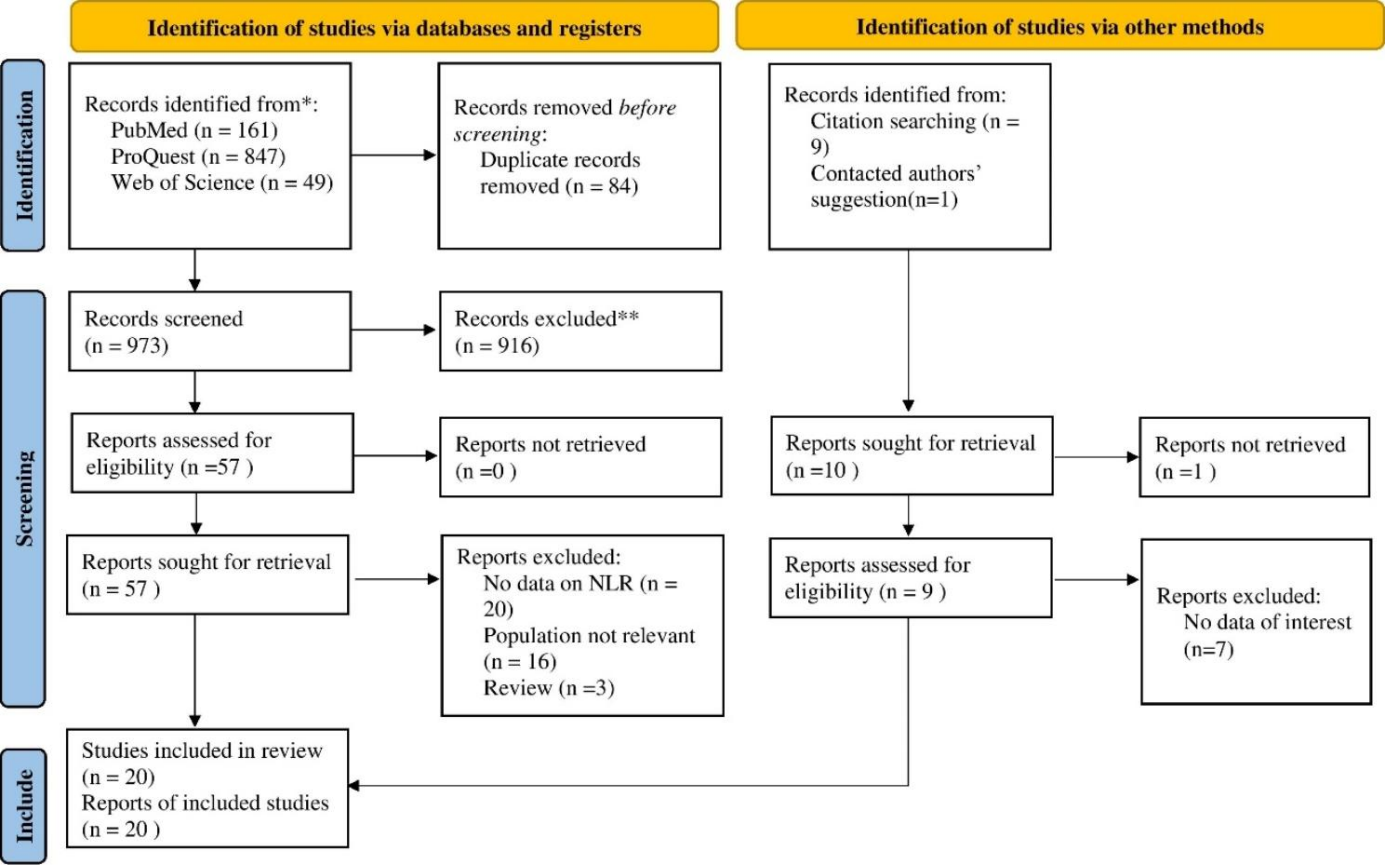
PRISMA 2020 Flow diagram for new systematic reviews which includes searches of databases, registers and other sources

There were 1067 total results from the manual search of the article citation list and the database search. Eventually, after removing duplicates and irrelevant records, 20 studies were included to this review [25–43, 47]. The PRISMA flow diagram, which illustrates the inclusion and exclusion procedure, appears in Fig. 1.

### Characteristics of the population and quality assessment

Totally, 20 articles were included in the analysis, comprising 3584 patients with PD and 2487 healthy controls [25–43, 47]. The general features of the included articles are shown in Table 1. According to the NOS scale, the quality assessment revealed that all of the studies were of moderate to high quality (Table 1).

**Table 1:** General characteristics of included studies

Author	Year	Design	Ethnicity	Parkinson’ disease		Healthy control		SEN	SP	NOS score
				N	NLR	N	NLR			
Akil	2014	Prospective	Caucasian	51	3.10 ± 1.30	50	2.10 ± 0.32	73	74	7
Ucar	2016	Retrospective	Caucasian	46	2.66 ± 1.05	20	2.46 ± 1.04			7
Moghaddam	2018	Prospective	Caucasian	388	2.50 ± 0.90	148	2.20 ± 0.80			6
Pekel	2018	Prospective	Caucasian	17	2.32 ± 0.77	21	3.04 ± 1.63			8
Solmaz	2018	Not declared	Caucasian	101	3.07	60	2.17	65	75	7
Jiang,S.	2019	Retrospective	East Asian	116	1.87 ± 0.71	99	1.67 ± 0.61			8
Kenangil	2019	Not declared	Caucasian	94	2.20 ± 1.13	97	2.18 ± 0.96			8
Yazar	2019	Not declared	Caucasian	211	2.28 ± 0.55	200	1.71 ± 0.50			8
Jin	2020	Retrospective	Caucasian	183	2.91 ± 1.74	89	3.00 ± 1.50			7
Munoz-Delgado	2021	Retrospective	Caucasian	377	2.47 ± 1.10	355	1.98 ± 0.91			8
Liu	2021	Retrospective	East Asian	101	2.59 ± 0.87	97	2.02 ± 0.76	49	83	6
Kara	2021	Retrospective	Caucasian	100	2.40 ± 0.10	61	1.90 ± 0.10	70	62	8
Wang2	2021	Retrospective	East Asian	453	8.47 ± 2.74	436	9.03 ± 2.91			6
Contaldi	2022	Prospective	Caucasian	58	2.63 ± 1.15	58	2.23 ± 0.78	69	48	7
Jiang,L.	2022	Retrospective	Caucasian	125	2.09 ± 0.89	124	2.04 ± 0.77			7
Madetko	2022	Retrospective	Caucasian	98	2.26 ± 1.12	99	1.76 ± 0.71			8
Paul	2022	Prospective	Caucasian	562	3.02 ± 1.60	227	2.16 ± 1.20			8
Wang1	2022	Retrospective	East Asian	153	2.89 ± 0.15	36	2.00 ± 0.16			8
Xing	2022	Prospective	East Asian	69	2.83 ± 2.91	36	1.98 ± 0.86			7
Munoz-Delgado	2023	Retrospective	Caucasian	281	2.56 1.16	174	2.18 0.79			7
N:Number; NLR: Neutrophil to lymphocyte ratio										

### PD patients’ NLR levels

**Fig. 2:**
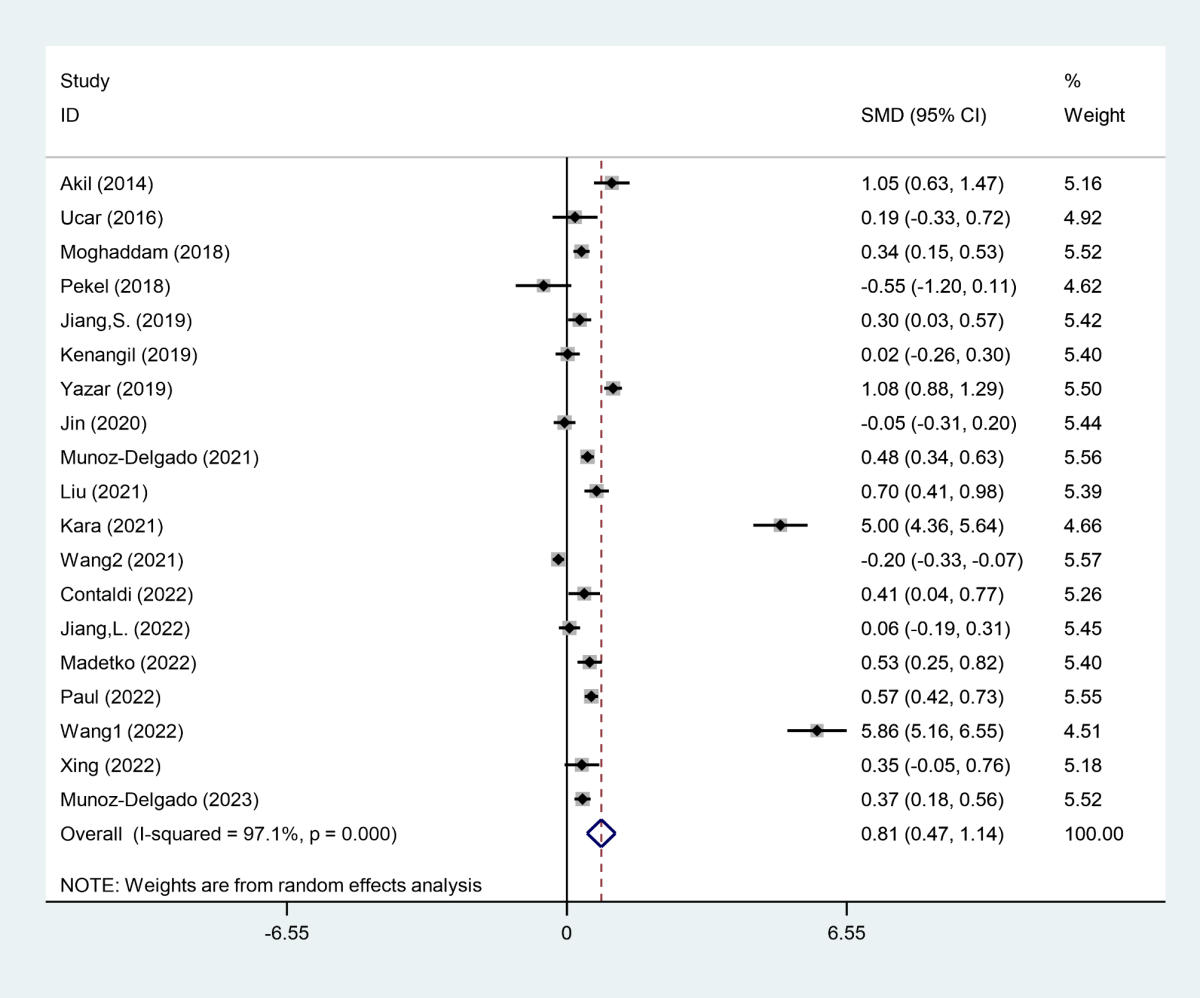
Meta-analysis of differences in NLR level between patients with PD and healthy controls

According to a random-effects model, NLR levels were higher in PD patients than in healthy controls (SMD = 0.81, 95% CI = 0.47 to 1.14, P < 0.001, Fig. 2). However, the certainty of evidence was very low (Table 2).

**Table 2:** GRADE^1^ Evidence Profile for cohort studies of the neutrophil to lymphocyte ratio in Parkinson’s disease

Certainty assessment							No of patients		Certainty	Importance
**№ of studies**	**Study design**	**Risk of bias** ^**2**^	**Inconsistency** ^**3**^	**Indirectness**	**Imprecision** ^**5**^	**Publication bias** ^**6**^	**Participants, n**	**Cases, n**		
20	observational studies	not serious	very serious	not serious	not serious	none	6071	3584	⨁◯◯◯ Very low	CRITICAL

^1^Grading of Recommendations Assessment, Development and Evaluation

^2^Risk of bias based on Newcastle-Ottawa Scale

^3^When I^2^ was < 30% inconsistency considered as Not serious limitation, > 50 considered as serious and more than 75% considered as very serious limitation

^5^Serious limitations when there was fewer than 400 participants for each outcome and very serious limitations when there was fewer than 300 participants for each outcome

^6^Funnel plot revealed no asymmetry; neither test of publication bias approached P < 0.10

**Fig. 3:**
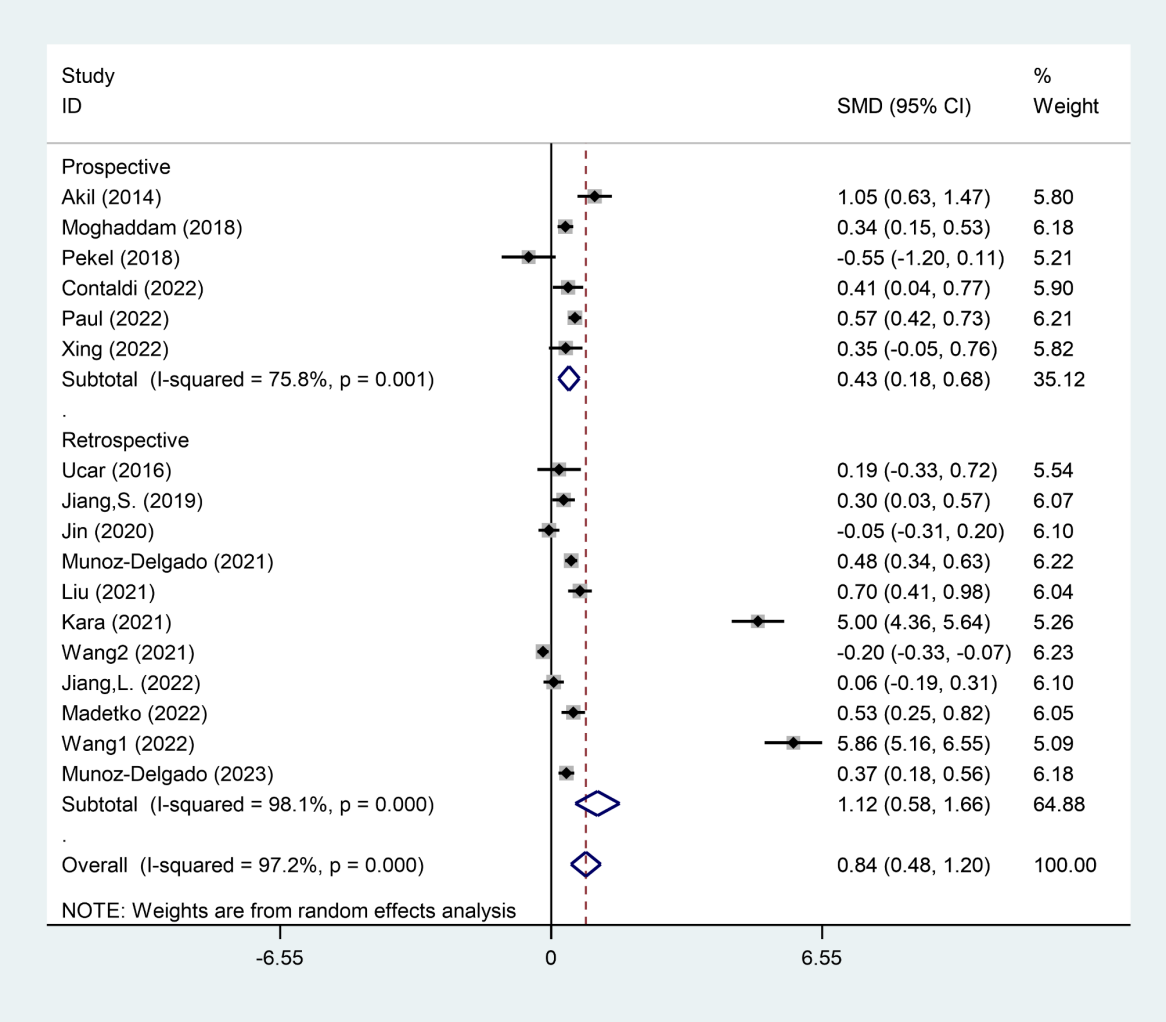
Subgroup analysis of differences in NLR level between patients with PD and healthy controls, according to study design

The results of subgroup analysis were shown in Table 3. In the subgroup analysis according to study design, we observed that PD patients exhibited increased levels of NLR in comparison to healthy controls in either retrospective (SMD = 0.93, 95% CI = 0.22 to 1.63, P < 0.010) or prospective (SMD = 0.43, 95% CI = 0.18 to 0.68, P = 0.001) studies (Fig. 3).

**Table 3:** Results of subgroup analysis

Shared characteristic	Subsets of participants	Number of studies	SMD	95%CI	P-value	I^2^	Cochran’s Q tests P-value
Study design	Retrospective	11	1.12	0.58 to 1.66	< 0.001	98.1	< 0.001
	Prospective	6	0.43	0.18 to 0.68	0.001	75.8	0.001
Ethnicity	East Asian	7	0.93	0.22 to 1.63	0.010	98.0	< 0.001
	Caucasian	12	0.75	0.40 to 1.10	< 0.001	95.7	< 0.001

**Fig. 4:**
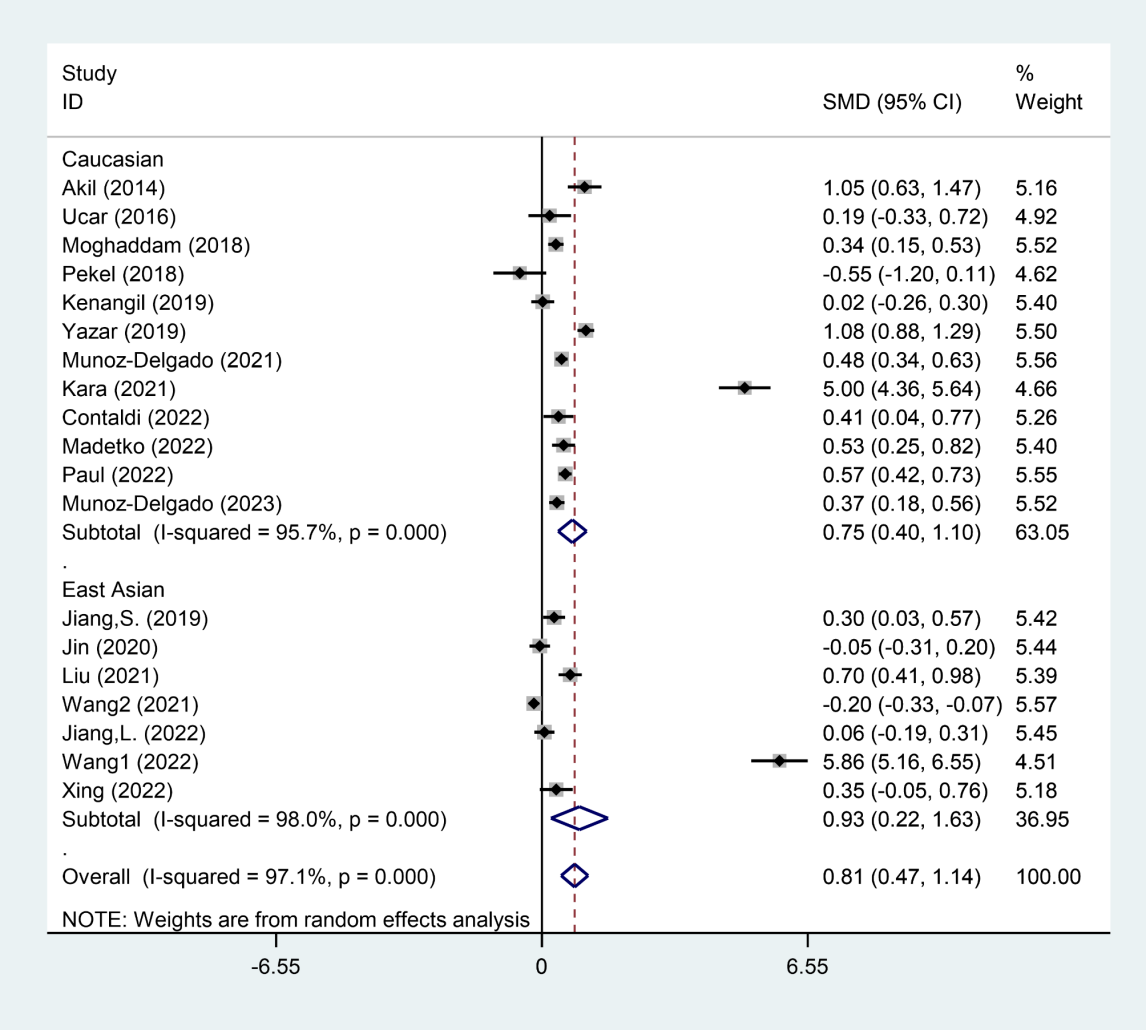
Subgroup analysis of differences in NLR level between patients with PD and healthy controls, according to ethnicity

In the subgroup analysis according to ethnicity, we observed that PD patients exhibited increased levels of NLR in comparison to healthy controls in either East Asian (SMD = 0.93, 95% CI = 0.22 to 1.63, P = 0.010) or Caucasian (SMD = 0.75, 95% CI = 0.40 to 1.10, P < 0.001) patients (Fig. 4).

### Sensitivity analysis

**Fig. 5:**
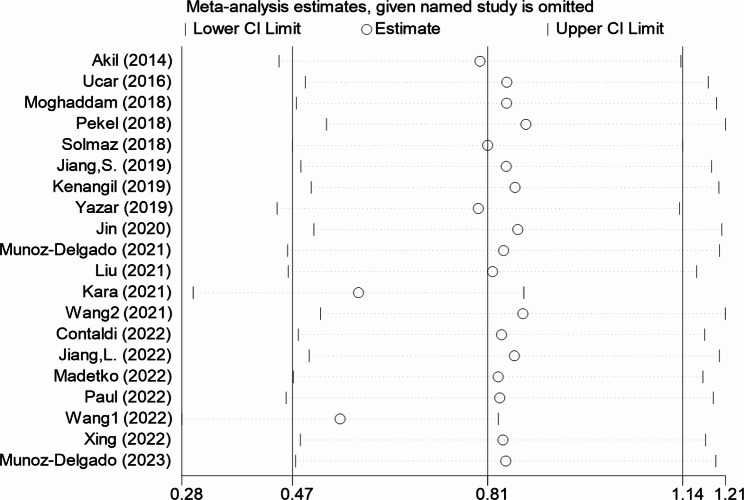
Sensitivity analysis

Our results were stable and not reversed after omitting each study in sensitivity analysis (Fig. 5, **Supplemental Table I**).

### NLR’s diagnostic value in PD

**Fig. 6:**
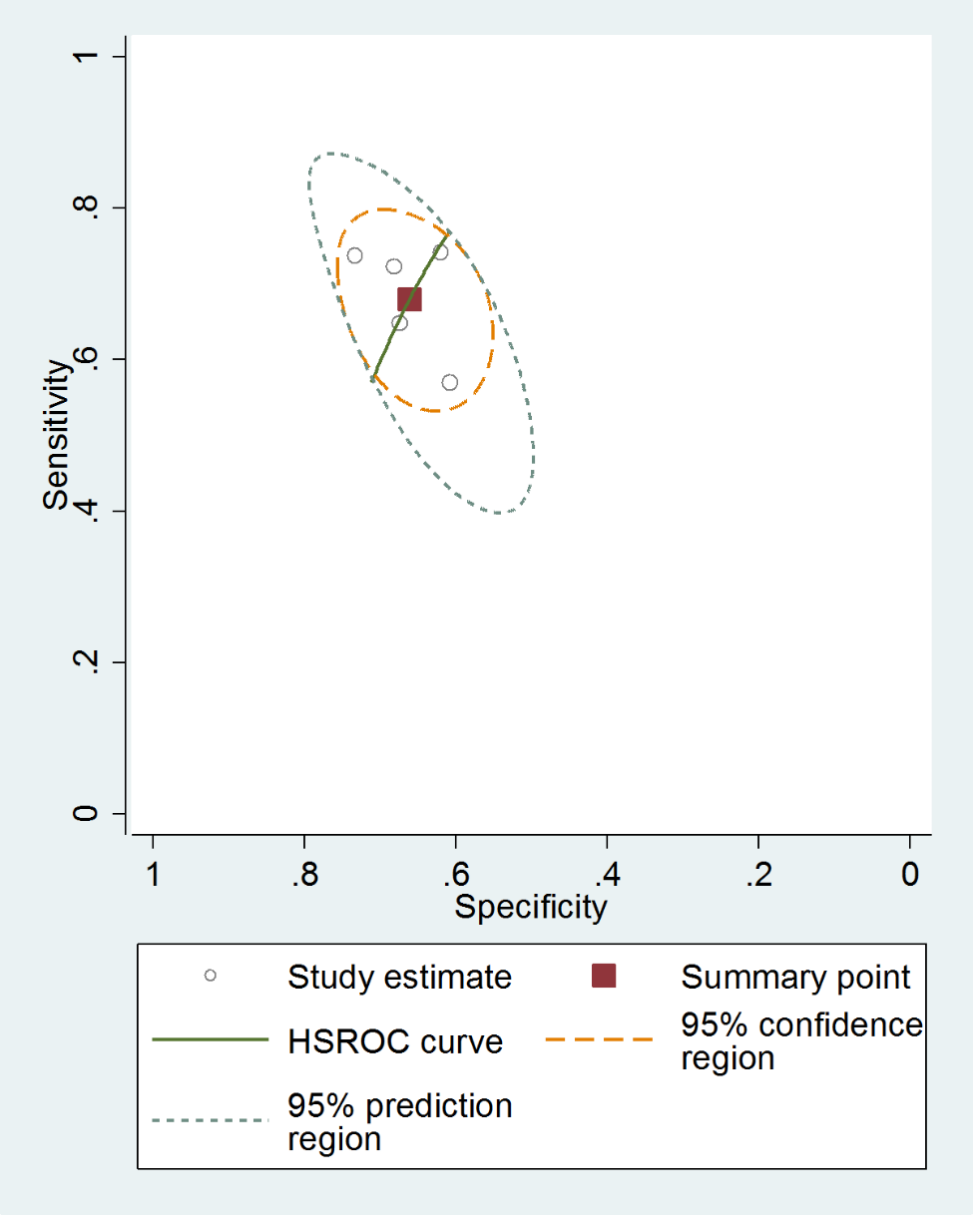
SROC curve of included studies assessing diagnostic value of NLR for PD

The pooled sensitivity was 0.67 (95% CI = 0.61–0.73), and the pooled specificity was 0.66 (95% CI, 0.61–0.70). The pooled positive likelihood ratio, negative likelihood ratio, diagnostic odds ratio (DOR) of NLR were 2.00(95%CI = 1.66–2.42), 0.48 (95%CI = 0.38–0.60), and 2.06 (95%CI = 1.65–2.58), respectively (Fig. 6).

### Publication bias

There were some signs of publication bias among the included articles (Egger test 0.001, Supplemental Figure I).

## Discussion

We undertook meta-analysis of 20 studies to evaluate the correlation of NLR with PD. It was discovered that PD patients had higher levels of NLR than healthy controls.

PD is a progressive neurodegenerative illness defined pathologically by emergence of lewy bodies and dopaminergic neuron loss in the substantia nigra. Neuroinflammation has a critical role in lewy body formation and substania nigra cell loss. Both the adaptive and innate immune systems are significantly altered in PD [48]. Inflammation is silent in the brain. The response relies on the production of inflammatory components by microglia in the brain. Microglia produce inflammatory cytokines that appear to play significant roles [49]. Interleukin-1 (IL-1), IL-6, and Tumor necrosis factor-alpha (TNF) are examples of inflammatory cytokines that amplify and sustain immune responses and inflammation. It is interesting that PD patients’ CSF basal ganglia contain elevated levels of TNFα, IL-6, and IL-1b [50]. The BBB’s integrity may be lost as a result of the release of pro-inflammatory cytokines by microglia and also upregulation of adhesive molecules (VCAM and ICAM), which cause infiltration of peripheral immune cell in brain [51]. The NLR is a low-cost, simple-to-use indicator of peripheral inflammation [52, 53]. Increased NLR has been shown in many CNS conditions [54]. Several studies evaluated association of increased NLR with PD and our meta-analysis confirmed this association. This may show important role of inflammation and it may exist several years before clinical manifestation of PD and the immune system involvement is promising for therapeutic targeting. In addition to diagnostic utilities, NLR can be used as a prognostic factor in PD. For example, in 2022, Muñoz-Delgado et al. conducted a retrospective cohort study on 211 PD patients as primary-cohort. For replication goals, they also included 344 separate patients with PD in Parkinson’s Progression Markers Initiative group as PPMI-cohort. They showed that NLR is associated with nigrostriatal dopaminergic system degeneration, assessed using striatal dopamine transporter (DAT) density. Patients with higher levels of NLR had significantly lower DAT levels in the putamen (primary-cohort: P = 0.02; PPMI-cohort: P = 0.02) and the caudate (primary-cohort: P < 0.001; PPMI-cohort: P = 0.05) [55]. In addition, Song et al. prospectively followed up 26,210 participants for 21 years. During the follow-up, 486 incidences of PD were reported. The results showed that NLR (HR = 1.09; 95% CI: 1.00- 1.19), was related to an increased risk of PD [56].

Although the role of chronic inflammation is confirmed in PD, it is unclear whether inflammation is the root cause of neurodegeneration or whether it develops as a consequence of cell degeneration and selective damage process [57]. Also the trigger factor for inflammation such as viral infection or autoimmunity remain to be elucidated. The role of inflammation may be different in various subtypes of PD. Use of anti-inflammatory, anti-viral drug and immune-modulating (immunotherapy)for PD treatment and even preventing of PD can be a new era for management of neurodegenerative disease [51, 58].

Due to the extensive evidence that inflammation and immune activation are characteristics of PD, it is not strange that immune-targeting interventions and anti-inflammatory drugs have progressed in the treatments for PD, as they have in other neurodegenerative illnesses such as amyotrophic lateral sclerosis and Alzheimer’s disease [59, 60]. Trials on anti-inflammatory medication in PD have been profoundly unsatisfactory, as they have been in other neurodegenerative illnesses, and such trials need adjusting the strategy [52]. The incorporation of immune-related end points might be a significant therapeutic factor that influences the design of future, possibly more effective trials that use combination medicines that target many processes, like immunological responses [52, 61].

As the neurodegenerative field grows, it is becoming more important to investigate the role of peripheral and central inflammation in the progression and pathogenesis of PD, one of the key obstacles is the creation of innovative methods, including advanced patient-derived models, machine learning, or single-cell multi-omics that will empower the field to thoroughly examine how immune cells predispose or protect neuron injury, whether or not inflammation and the immune system are significant elements of the disease, and at which stages immune processes play crucial roles and are prognostic of illness course [62, 63]. Therefore, further research in these areas is required. Better knowledge and identification of the immunological processes behind the initial indications of PD may result in innovative medicines, and physicians may one day be able to successfully intervene with repurposed or novel immunomodulatory and anti-inflammatory medications to postpone or slow progression of the illness from the periphery to the CNS [64].

There were several limitations to this study. First, only summary data, rather than individual patient data, can be utilized. Second, we only considered studies that reported mean and SD. Third, there was a significant publication bias in our results. Forth, we could not perform trial sequential analysis (TSA), because it can be applied to analyses on dichotomous data like odds ratio (OR) and hazard ratio (HR), but not on SMD, which was reported in our meta-analysis. We tried to extract dichotomous data like OR from included studies and conduct a new meta-analysis and then, perform TAS; however, except to one study [33], no study had reported OR, so further studies reporting TSA results are needed. Finally, neutrophil and lymphocyte counts are nonspecific measures that might be affected by other circumstances such as infections, inflammation, and medicines. These contemporaneous variables may affect how NLR is measured in some of the included studies since they were not specifically controlled.

## Conclusion

In conclusion, high NLR can be regarded as an indicator of inflammation in PD. Still, it needs more investigations to study its diagnostic value, association with the stage and progression of the disease, and therapeutic value. Our results expanded immunological information from Parkinson’s disease patients to build better preclinical models, with the long-term objective of allowing early detection of at-risk people to postpone, and treat the illness more properly.

### Electronic supplementary material

Below is the link to the electronic supplementary material.

Supplementary Material 1

## Data Availability

The full text of this article also contains the dataset used to support its findings.

## List of Abbreviations

PD: Parkinson’s disease
TNFs: Tumor necrosis factors
NLR: Neutrophil to lymphocyte ratio
CBC: Complete blood count
PRISMA: Preferred Reporting Items for Systematic reviews and Meta-Analyses
SD: Standard deviation
NOS: Newcastle-Ottawa scale
SMD: Standardized mean difference
CI: Confidence interval
DOR: Diagnostic odds ratio
SROC: Summary receiver operating characteristic
IL: Interleukin
