# Supplementary material for: Neutrophil to lymphocyte ratio in parkinson’s disease: a systematic review and meta-analysis
Source: BMC Neurol. 2023 Sep 21;23:333. doi: 10.1186/s12883-023-03380-7 (PMC10512499; doi:10.1186/s12883-023-03380-7)
Supplement: Supplementary file 1 — Supplementary Material 1 [file 12883_2023_3380_MOESM1_ESM.docx]

**Neutrophil to lymphocyte ratio in Parkinson’s disease: A systematic review and meta-analysis**

| **Supplemental Table I.** Results of sensitivity analysis | | |
| --- | --- | --- |
| Omitted study | SMD | 95%CI |
| Akil | 0.79 | 0.45 to 1.13 |
| Ucar | 0.83 | 0.49 to 1.18 |
| Moghaddam | 0.83 | 0.48 to 1.19 |
| Pekel | 0.87 | 0.53 to 1.21 |
| Solmaz | 0.80 | 0.47 to 1.13 |
| Jiang,S. | 0.83 | 0.48 to 1.18 |
| Kenangil | 0.85 | 0.50 to 1.19 |
| Yazar | 0.78 | 0.44 to 1.13 |
| Jin | 0.85 | 0.50 to 1.20 |
| Munoz-Delgado | 0.83 | 0.46 to 1.20 |
| Liu | 0.81 | 0.46 to 1.16 |
| Kara | 0.58 | 0.30 to 0.86 |
| Wang2 | 0.86 | 0.52 to 1.21 |
| Contaldi | 0.82 | 0.48 to 1.17 |
| Jiang,L. | 0.85 | 0.50 to 1.20 |
| Madetko | 0.82 | 0.47 to 1.17 |
| Paul | 0.82 | 0.46 to 1.19 |
| Wang1 | 0.55 | 0.28 to 0.82 |
| Xing | 0.83 | 0.48 to 1.17 |
| Munoz-Delgado | 0.83 | 0.47 to 1.19 |
| SMD: Standardized mean difference; 95% CI: 95% confidence interval | | |

**Supplemental Figure I.** Funnel plot assessing publication bias
